# Supplementary figures and images for: Impact of deep brain stimulation of the subthalamic nucleus on natural language in patients with Parkinson’s disease
Source: PLoS One. 2020 Dec 29;15(12):e0244148. doi: 10.1371/journal.pone.0244148 (PMC7771859; doi:10.1371/journal.pone.0244148)

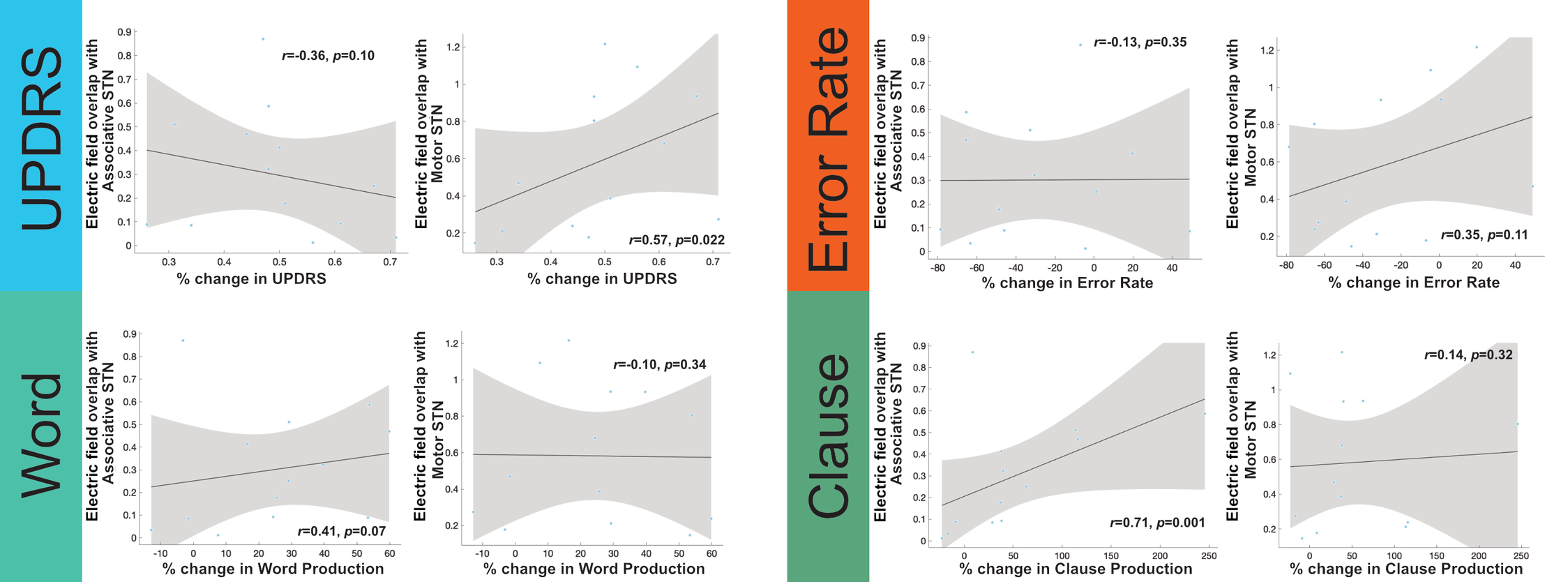

Supplement: S1 Fig — Spearman correlations between the intersection of the electric field with each STN subregion (combined for both hemispheres) and changes in UPDRS, word and clause production rates, and error rate indicated A) a positive correlation between the improvement in the UPDRS and the electric field overlap with the motor STN, but not with the associative STN (top left), B) an association between the increase in the word production rate and the electric field overlap with the associative STN (on the level of trend), but not the motor STN (bottom left), and C) a positive correlation between the increase in the clause production rate and the electric field overlap with the associative STN, but not with the motor STN (bottom right). D) changes in error rates were neither significantly related to the electric field overlap with the associative, nor to the motor STN (top right). (TIF) [file pone.0244148.s001.tif]
