# Supplementary material for: Impact of deep brain stimulation of the subthalamic nucleus on natural language in patients with Parkinson’s disease
Source: PLoS One. 2020 Dec 29;15(12):e0244148. doi: 10.1371/journal.pone.0244148 (PMC7771859; doi:10.1371/journal.pone.0244148)
Supplement: S1 Table — Overview of the localization of active electrodes in the xyz-space; due to missing post-operative MRI the data from participant no. 8 did not enter the localization analysis. Electrode localizations of all 26 active electrodes from the thirteen participants are depicted in Fig 1. Pt.: participant code. (DOCX) [file pone.0244148.s002.docx]

**S1 Table. Electrode localization in the XYZ-space**

|  | **Left** | | | **Right** | | |
| --- | --- | --- | --- | --- | --- | --- |
| **Pt.** | **X** | **Y** | **z** | **x** | **y** | **z** |
| 1 | -12.657 | -10.367 | -8.929 | 10.972 | -12.355 | -8.541 |
| 2 | -9.646 | -15.017 | -8.553 | 8.530 | -15.924 | -9.064 |
| 3 | -11.853 | -14.750 | -7.337 | 11.421 | -14.316 | -5.959 |
| 4 | -12.220 | -13.887 | -7.316 | 11.350 | -13.801 | -6.908 |
| 5 | -11.179 | -11.461 | -7.280 | 11.434 | -11.292 | -6.414 |
| 6 | -11.698 | -13.637 | -5.218 | 10.426 | -13.229 | -5.240 |
| 7 | -11.749 | -13.211 | -7.369 | 11.125 | -13.097 | -7.351 |
| 9 | -14.052 | -11.927 | -4.184 | 13.297 | -12.160 | -5.003 |
| 10 | -12.555 | -14.320 | -6.680 | 11.755 | -13.202 | -7.705 |
| 11 | -12.775 | -12.808 | -5.003 | 11.989 | -12.542 | -10.661 |
| 12 | -11.354 | -13.651 | -7.707 | 10.399 | -13.570 | -7.575 |
| 13 | -12.117 | -15.616 | -7.505 | 11.466 | -15.146 | -8.697 |
| 14 | -12.460 | -12.915 | -5.591 | 11.760 | -12.369 | -5.961 |
