# Supplementary material for: Impact of deep brain stimulation of the subthalamic nucleus on natural language in patients with Parkinson’s disease
Source: PLoS One. 2020 Dec 29;15(12):e0244148. doi: 10.1371/journal.pone.0244148 (PMC7771859; doi:10.1371/journal.pone.0244148)
Supplement: S2 Table — Overview of all semantic (A) and syntactic (B) stylistic devices analyzed, including the overall type and subcategories. (DOCX) [file pone.0244148.s003.docx]

**S2 Table. Stylistic devices**

| **A. Semantic stylistic devices** | | | | | |  |
| --- | --- | --- | --- | --- | --- | --- |
|  | **Subcategory** | | | **Sub-subcategory** | | **Sub-subcategory** |
|  |  | | |  | |  |
| **Addition** | Correction | | | Correction | |  |
|  |  | | | Retraction | |  |
|  | Polysyndeton | | |  | |  |
|  | Epitheton | | |  | |  |
|  | Paraphrase | | |  | |  |
|  | Praeteritio | | |  | |  |
|  | Redundancy, broader sense | | | Pleonasm | |  |
|  |  | | | Gemination | |  |
|  |  | | | Tautology | |  |
|  |  | | | Perissologia | |  |
|  |  | | | Redundancy, narrower sense | |  |
| **Omission** | Elision | | | Apheresis | |  |
|  |  | | | Apocope | |  |
| **Transposition** | Archaisms | | |  | |  |
|  | Hyperlage | | |  | |  |
|  | Anachronism | | | Backward | |  |
|  |  | | | Forward | |  |
| **Permutation** | Antiphrasis | | |  | |  |
|  | Hyperbola | | |  | |  |
|  | Understatement | | |  | |  |
|  | Catachresis | | |  | |  |
|  | Paradox | | | Contradictio in adiecto | |  |
|  |  | | | Oxymoron | |  |
|  | Exemplum | | | Current example | |  |
|  |  | | | Historic example | |  |
|  |  | | | Poetic example | |  |
|  | Tropes | | | Antonomasia | |  |
|  |  | | | Allegory | |  |
|  |  | | | Symbol | |  |
|  |  | | | Metonymy / Synecdoche | | Pars pro toto |
|  |  | | |  | | Toto pro pars |
|  |  | | | Mataphor | | Euphemism |
|  |  | | |  | | Anthropomorhism |
|  |  | | |  | | Other metaphor |
|  |  | | | Irony | | Litotes |
|  |  | | |  | | Sarcasm |
|  |  | | |  | | Cynicism |
|  |  | | |  | | Other irony |
| **Other** | Peripeteia | | |  | |  |
|  | Pejorative | | |  | |  |
|  | Alogism | | |  | |  |
|  | Neologism | | |  | |  |
|  | Direct speech | | |  | |  |
|  | Indirect speech | | |  | |  |
| **B.** **Syntactic stylistic devices** | | | | |  |  |
|  | | **Category** | **Subcategory** | |  |  |
|  | |  |  | |  |  |
| **Addition** | | Epiphrasis |  | |  |  |
|  | | Parenthesis |  | |  |  |
|  | | Enumeration |  | |  |  |
| **Omission** | | Ellipsis | Aposiopesis | |  |  |
|  | |  | Brachyology | |  |  |
|  | |  | Apokoinu construction | |  |  |
|  | |  | Other ellipsis | |  |  |
| **Transposition** | | Hyperbaton |  | |  |  |
|  | | Hysteron-proteron |  | |  |  |
|  | | Inversion |  | |  |  |
| **Other** | | Rhetorical question |  | |  |  |

Overview of all semantic (A) and syntactic (B) stylistic devices analyzed, including the overall type and subcategories.
