# Supplementary material for: Impact of deep brain stimulation of the subthalamic nucleus on natural language in patients with Parkinson’s disease
Source: PLoS One. 2020 Dec 29;15(12):e0244148. doi: 10.1371/journal.pone.0244148 (PMC7771859; doi:10.1371/journal.pone.0244148)
Supplement: S3 Table — Distribution of language error rates (number of errors per total word count) across categories and participants. (DOCX) [file pone.0244148.s004.docx]

**S3 Table. Stylistic devices**

|  | **DBS ON** |  | **DBS OFF** |  |
| --- | --- | --- | --- | --- |
| Errors per total word count | Mean | *SD* | Mean | *SD* |
| Grammatical | 0.050 | *0.034* | 0.063 | *0.034* |
| Lexical | 0.016 | *0.012* | 0.016 | *0.015* |
| phonetic | 0.005 | *0.012* | 0.004 | *0.008* |
| contextual | 0.000 | *0.000* | 0.000 | *0.000* |
| Stylistic | 0.020 | *0.023* | 0.050 | *0.043* |
| idiomatic | 0.002 | *0.003* | 0.001 | *0.003* |
| Pragmatic | 0.025 | *0.020* | 0.020 | *0.019* |
| Logic | 0.004 | *0.005* | 0.006 | *0.012* |
